# Thermal Damage-Free Microwave Annealing with Efficient Energy Conversion for Fabricating of High-Performance a-IGZO Thin-Film Transistors on Flexible Substrates

**DOI:** 10.3390/ma14102630

**Published:** 2021-05-18

**Authors:** Ki-Woong Park, Won-Ju Cho

**Affiliations:** Department of Electronic Materials Engineering, Kwangwoon University, Kwangwoon-ro 20, Nowon-gu, Seoul 139-701, Korea; wwoong97@naver.com

**Keywords:** thermal damage free, microwave annealing, efficient energy conversion, flexible substrates, a-IGZO

## Abstract

In this study, we applied microwave annealing (MWA) to fabricate amorphous In-Ga-Zn-O (a-IGZO) thin-film transistors (TFTs) without thermal damage to flexible polyimide (PI) substrates. Microwave energy is highly efficient for selective heating of materials when compared to conventional thermal annealing (CTA). We applied MWA and CTA to a-IGZO TFTs on PI substrate to evaluate the thermal damage to the substrates. While the PI substrate did not suffer thermal damage even at a high power in MWA, it suffered severe damage at high temperatures in CTA. Moreover, a-IGZO TFTs were prepared by MWA at 600 W for 2 min, whereas the same process using CTA required 30 min at a temperature of 300 °C, which is a maximum process condition in CTA without thermal damage to the PI substrate. Hence, MWA TFTs have superior electrical performance when compared to CTA TFTs, because traps/defects are effectively eliminated. Through instability evaluation, it was found that MWA TFTs were more stable than CTA TFTs against gate bias stress at various temperatures. Moreover, an MWA TFT-constructed resistive load inverter exhibited better static and dynamic characteristics than the CTA TFT-constructed one. Therefore, MWA is a promising thermal process with efficient energy conversion that allows the fabrication of high-performance electronic devices.

## 1. Introduction

With the remarkable advances in display technology, flexible or foldable electronic devices are attracting considerable attention. Therefore, extensive studies have been conducted on flexible thin-film transistors (TFTs) for backplane driving devices and flexible substrates such as polymer plastics, ultra-thin glass (UTG) and metal foils [1,2,3,4]. Among various flexible substrates, polyimide (PI) is attracting much attention due to its low cost, relatively high thermal stability, and excellent mechanical/chemical properties [5,6,7]. Although PI is more suitable for high-temperature processes than other plastic substrates, it does suffer damage at high temperatures due to the inherent thermal limitations of plastic substrates [8,9]. Thus, stringent restrictions are placed on the thermal process, which is essential for improving device performance in flexible substrate-based electronic devices. For the fabrication of flexible and transparent TFTs, oxide semiconductor materials such as amorphous Indium-Gallium-Zinc-Oxide (a-IGZO), are receiving increasing attention due to their excellent electrical and optical characteristics, mechanical endurance, and chemical and thermal stability [10,11,12,13]. The conventional thermal annealing (CTA) process using an electrical resistance heating furnace has the advantages of low cost and high wafer throughput, and it is mainly used in post-deposition annealing (PDA) after depositing oxide semiconductors. However, because this convection heating process requires a long time at high temperatures, it may cause serious thermal damage to the flexible substrate and consequent deterioration of device performance [14,15]. Therefore, a technology for efficient PDA is essential for improving the electrical properties of a device without causing thermal damage to the flexible substrate.

In this study, we present the fabrication of thermal damage-free a-IGZO TFTs on flexible and transparent PI substrates using microwave annealing (MWA). MWA is a method in which materials couple with microwaves, absorb the electromagnetic energy volumetrically, and transform into heat, which differs from CTA, in which heat is transferred between objects by conduction, radiation, and convection mechanisms. Compared to conventional heating technology, microwave heating has additional advantages such as higher heating rates, energy transfer as opposed to heat transfer, no direct contact between the heating source and the heated material, material selective heating, volumetric heating, quick start-up and stopping, greater control of the heating process, significant savings in energy consumption, compactness, low cost and maintainability [16,17,18]. The selective heating capability of the material enables annealing of only the a-IGZO channels without thermal damage to the PI substrate, which is transparent to microwaves. This is a distinct advantage that MWA has over CTA in furnace, and it is highly suitable as a heat treatment method for flexible insulator substrates that have weak heat resistance such as PI. Although several studies have been conducted on the application of microwave to a-IGZO-TFTs to date [19,20,21], there are few reports of microwave annealing effects on improved performance and substrate heat damage of a-IGZO-TFT formed on flexible substrates. Therefore, this work aims to establish MWA as the cornerstone of fabrication processes for flexible electronics.

**Figure 1:**
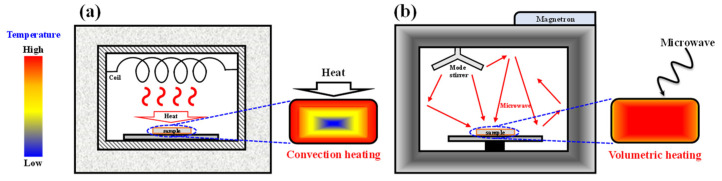
(**a**) Schematic of annealing equipment and the temperature profile inside the samples for (**a**) Conventional thermal annealing (CTA) furnace and (**b**) Microwave annealing (MWA) furnace.

Figure 1 exhibits a schematic of annealing equipment and the temperature profile inside the samples for (a) CTA furnace and (b) MWA furnace. MWA was applied to fabricate a-IGZO TFTs with a bottom-gate top-contact configuration on transparent and flexible PI substrates. For comparison, the same device structures were also prepared by CTA. The thermal damage of transparent, flexible PI substrates and the electrical properties of the a-IGZO TFTs were evaluated. In addition, the instability of a-IGZO TFTs was measured using the positive gate bias temperature stress (PBTS) and negative gate bias temperature stress (NBTS) tests. Then, resistive load inverter circuits were constructed by connecting a resistor in series with a-IGZO TFT and the static and dynamic characteristics were measured. Through the investigation of the deterioration of the PI substrates and the performance evaluation of the fabricated device and inverter circuit, the distinct strengths of MWA over CTA became clear.

## 2. Materials and Methods

The PI substrate was subjected to heat treatment under various microwave powers and furnace temperatures to evaluate and compare the thermal damage caused by MWA and CTA, respectively. For the heat-treated PI substrates, the critical process conditions for MWA or CTA were investigated by measuring the transmittance using UV–vis spectroscopy and evaluating the durability using the substrate bending test. To prepare the flexible substrates, 6 μm-thick PI was spin-coated on rigid glass. Then, a 100/100 nm-thick SiN_x_/SiO_2_ protective layer was deposited by a plasma-enhanced chemical vapor deposition system to a prevent chemical attack on the PI substrate and to ensure firm adhesion of the Al bottom gate electrode and gate insulator to the flexible PI substrate. The bottom gate electrode of a-IGZO TFTs was formed by depositing a 150 nm-thick Al film with an E-beam evaporator, patterning by photolithography, and then wet etching with H_3_PO_4_ solution. Subsequently, a 130 nm-thick SiO_2_ gate insulator was deposited by an RF magnetron sputter at a working pressure of 4.0 mTorr, RF power of 200 W, and Ar/O_2_ flow rate of 30/2 sccm. For the formation of the active channel layer, a 50 nm-thick a-IGZO (In_2_O_3_:Ga_2_O_3_:ZnO = 4:2:4.1 mol.%) film was deposited by an RF magnetron sputter at a working pressure of 6.0 mTorr, RF power of 100 W and Ar flow rate of 30 sccm, followed by photolithographic patterning and 30:1 buffered oxide etchant (BOE) wet etching. Finally, the source and drain (S/D) electrodes of a-IGZO TFTs were formed by a lift-off process after depositing a 150 nm-thick Ti film with an E-beam evaporator. The fabricated channel width and length were 20 μm and 10 μm, respectively. The PDA process for improving the electrical properties of a-IGZO TFT was performed using both MWA and CTA. For MWA, a microwave irradiation system with a frequency of 2.45 GHz was used. A resistance heating furnace was used for CTA. The conditions of PDA were determined using the CTA temperature at which the PI substrate showed no evidence of optical or mechanical damage, and the MWA power corresponding the equivalent sample heating effect. In order to test the PI substrates in similar temperature conditions, we accurately measured the sample temperature in the MWA with an infrared (IR) thermometer and then adjusted the microwave power to correspond to the CTA temperature. The PDA conditions of MWA and CTA presented in this study were 600 W for 2 min and 300 °C for 30 min, respectively, and both were annealed in ambient air.

**Figure 2:**
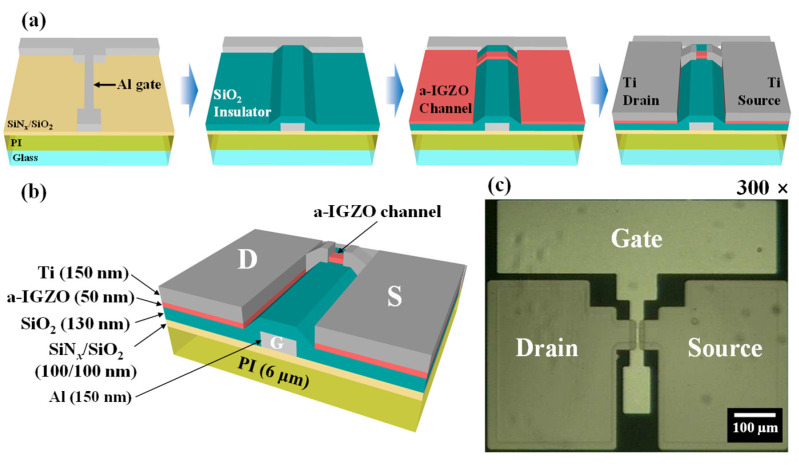
Schematic of (**a**) fabrication process, (**b**) completed structure of back-gate top-contact amorphous In-Ga-Zn-O (a-IGZO) thin-film transistors (TFT). (**c**) Optical microscope image of top view of the fabricated IGZO TFT.

Figure 2a,b show a schematic of the fabrication process sequence and completed structure of back-gate top-contact a-IGZO TFT. Figure 2c exhibits an optical microscope image of the fabricated TFT. The effects of the PDA on the electrical characteristics (such as field-effect mobility, subthreshold swing, interface trap density, and on/off current ratio) were measured with an Agilent 4156B Precision Semiconductor Parameter Analyzer. Additionally, threshold voltage (V_TH_) shifts were monitored using positive/negative gate bias temperature stress (PBTS/NBTS) tests at various temperatures to evaluate reliability and instability. In addition, resistive load inverter circuits were constructed by connecting a 400 MΩ resistor in series with a-IGZO TFTs and their static and dynamic characteristics were studied to evaluate their application potential. Electrical measurements of the a-IGZO TFTs and inverter circuit were performed in a shielded dark box to avoid external influences such as light and electrical noise.

## 3. Results and Discussion

**Figure 3:**
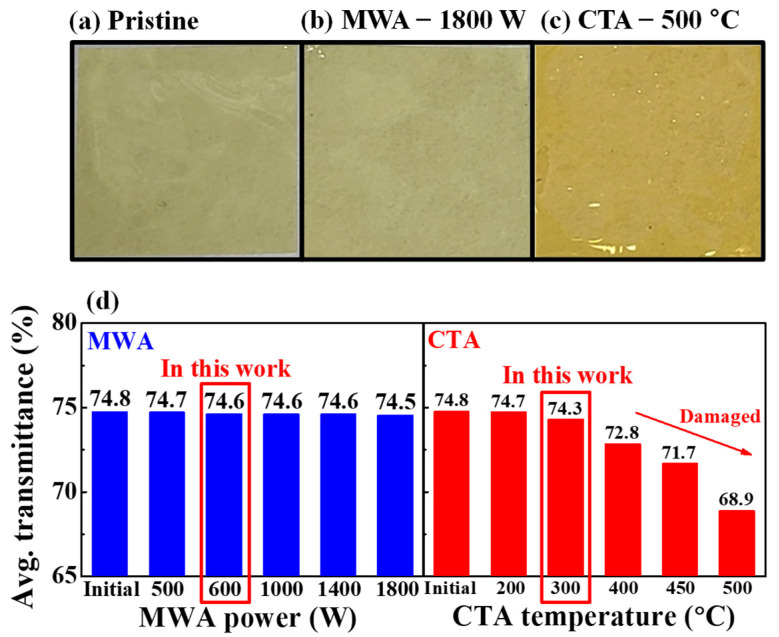
Photographic images of (**a**) pristine, (**b**) MWA (1800 W, 2 min) and (**c**) CTA processed (500 °C, 30 min) PI substrates. (**d**) Average transmittance of the PI substrates in the visible light region.

Figure 3 shows photographic images and average transmittance of the pristine, MWA and CTA processed flexible PI substrates. By comparing the photographic images, it can be seen that the MWA processed PI substrate with 1800 W for 2 min depicted in Figure 3b experienced little change from the pristine PI substrate depicted in Figure 3a, whereas the CTA processed PI substrate with 500 °C for 30 min depicted in Figure 3c has undergone a change in color. Figure 3d shows the average transmittance of the PI substrate in the visible light region. It was found that the optical properties of PI substrates depend on the PDA method and conditions. In the MWA, the transmittance of the PI substrate remained almost constant, regardless of the increase in microwave power, whereas in the CTA, it began to decrease from 400 °C. Thus, it was determined that the critical process temperature of CTA allowed for the PI substrate in this study is 300 °C [22]. The average transmittance in the visible region was 74.8%, 74.5%, and 68.9% for the pristine state, MWA at 1800 W, and CTA at 500 °C, respectively. This difference is because the PI substrate, which is an insulator, is almost transparent to microwaves and hardly suffers thermal damage. Therefore, it is concluded that MWA has distinct advantages over CTA and is a highly suitable annealing method for thermally vulnerable substrates.

**Figure 4:**
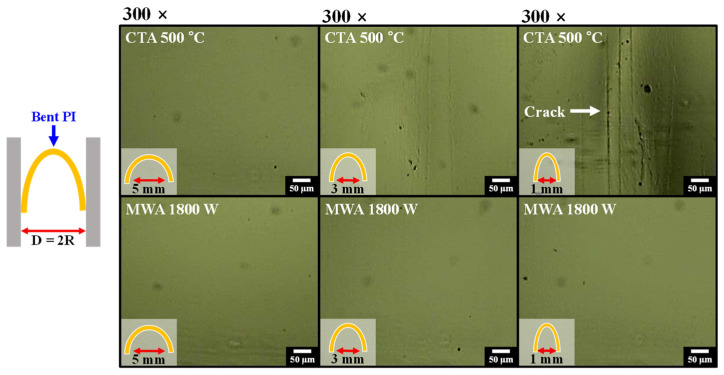
Optical microscopic images of CTA (upper images) and MWA processed (lower images) PI substrates after bending them 100 times.

Figure 4 shows the optical microscope images of flexible PI substrates after the bending test. As mechanical strength is an important characteristic for flexible substrates, we evaluated the bending endurance of the PI substrates processed by CTA (500 °C, 30 min) and MWA (1800 W, 2 min). The simplest and most commonly used bending radius test method was adopted. The bending radii (R = D/2) of 2.5, 1.5, and 0.5 mm were selected, which were sufficiently tough conditions to test the PI substrate. For every bending radius, 100 bending motions were repeated. In the case of CTA (upper images of Figure 4), due to thermal damage of the PI substrate by convection heating, the PI substrate creased at the 1.5 mm-bending radius and fatally cracked at the 0.5 mm-bending radius. By contrast, the PI substrate processed by MWA (bottom images of Figure 4), showed remarkable bending durability even at a 0.5 mm bending radius compared to CTA. From the results in Figure 3 and Figure 4, it is found that CTA directly induces thermal damage to the PI substrate, reducing optical and mechanical properties. In contrast, MWA is more suitable for annealing a-IGZO TFTs without damaging the transparent and flexible PI substrate, due to its selective heating capability.

**Figure 5:**
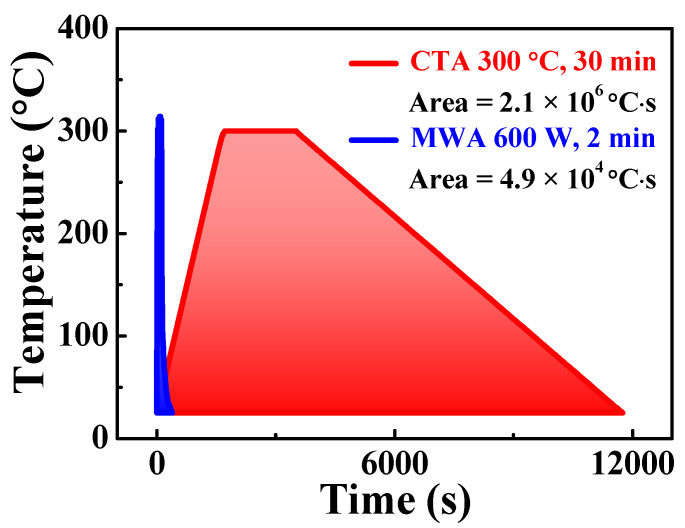
Temperature profile and heat budget for CTA (300 °C, 30 min) and MWA (600 W, 2 min) processes. MWA features faster ramp-up and ramp-down, and shorter processing time.

Figure 5 shows the temperature profile and thermal budget of the PDA processes for fabricating a-IGZO TFTs on flexible PI substrates. The temperature of CTA at 300 °C was chosen to minimize/avoid thermal damage to the PI substrate. Then, using an IR thermometer, the sample temperatures were accurately measured in MWA, and the microwave power was determined to be 600 W, which is the condition in which the sample temperature is equivalent to the CTA temperature of 300 °C. Namely, these conditions are intended to exclude differences in sample heating temperature, and to include only differences in heating methods when evaluating the characteristics of TFTs. The thermal budgets calculated by integrating the temperature–time curves are 2.1 × 10^6^ °C·s and 4.9 × 10^5^ °C·s for CTA and MWA respectively, revealing that the MWA has a thermal budget 40 times lower than the CTA. This large difference is due to differences in ramp-up and ramp-down, and main process times. With efficient energy conversion, MWA delivers microwave energy directly to the materials, and features volumetric heating and quick start-up and stop, enabling relatively short processing times for PI substrates with poor thermal durability.

**Figure 6:**
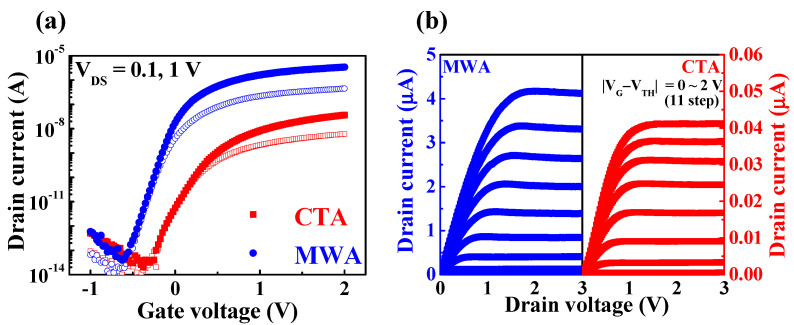
(**a**) Transfer and (**b**) output characteristics of CTA (300 °C, 30 min) and MWA processed (600 W, 2 min) a-IGZO TFTs on flexible polyimide (PI) substrates.

Figure 6 shows the electrical characteristics of flexible a-IGZO TFTs on PI substrates processed by MWA (600 W, 2 min) and CTA (300 °C, 30 min). The transfer characteristics (I_D_-V_G_) in Figure 6a were measured by sweeping the gate voltage (V_G_) from −1 to 2 V at a drain voltage (V_D_) of 0.1 and 1 V. Compared to CTA TFT, the on-current of MWA TFT is two orders of magnitude higher and the drain current (I_D_) also increases more steeply with an increase in V_G_. The V_TH_ of the CTA TFT and MWA TFT are 0.23 and −0.10 V, respectively. Figure 6b shows the output characteristics (I_D_-V_D_), where I_D_ increases linearly at low V_D_ regions, showing clear pinch-off and saturation behavior at high V_D_ regions. It can be seen that MWA TFT has a much higher driving current than CTA TFT.

**Figure 7:**
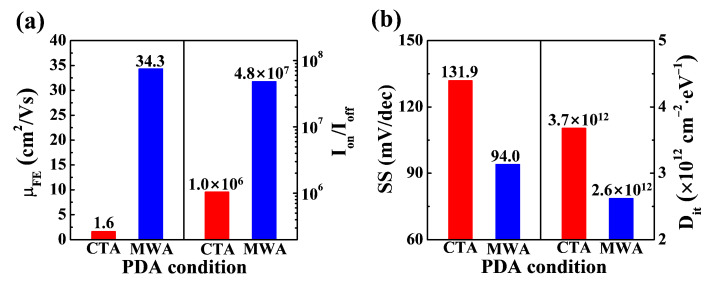
Electrical parameters of a-IGZO TFTs on flexible PI substrate for CTA (300 °C, 30 min) and MWA processing (600 W, 2 min). (**a**) Field effect mobility and on/off current ratio. (**b**) Subthreshold swing and interface trap density.

Figure 7 shows the electrical parameters of flexible a-IGZO TFTs on PI substrates prepared by MWA and CTA. In Field Effect Transistor (FET) devices, a large field effect mobility (*μ_FE_*) and small threshold swing (SS) are desirable for a fast-switching performance and low power consumption. The *μ_FE_*, which determines the switching speed, was calculated using the following equation:

(1) $$\mu_{FE}=\frac{Lg_{m}}{WC_{ox}V_{D}},\;g_{m}={\frac{\partial I_{D}}{\partial V_{G}}|}_{V_{D}=Const}$$

where *L*, *W*, *g_m_*, and *C_ox_* are the channel length, width, transconductance, and gate oxide capacitance per unit area, respectively. The *μ_FE_* of TFTs was extracted at *V_D_* = 0.1 V. The *μ_FE_* of a-IGZO TFTs on the PI substrate was 1.6 cm^2^/V·sec and 34.3 cm^2^/V·sec for the CTA and MWA processes, respectively. The increase in field effect mobility in the MWA process indicates that microwave annealing effectively contributed to the improvement of channel conductance in a short processing time without thermal damage to the PI substrate. The on/off current ratios (*I_on_/I_off_*) and the drain current ratios for when the transistor is turned on and off were 1.0 × 10^6^ and 4.8 × 10^7^ for the CTA and MWA processes, respectively. Meanwhile, a small *SS* is a major concern for a low-power operation, because it allows a lower V_TH_ for the same off current, enabling the driving of the transistor at a lower supply voltage. The *SS* of the FET is defined as the change in gate voltage to achieve a one-decade change in I_D_, and it can be calculated from the *I_D_–V_G_* curve using the following equation:

(2) $$SS=\frac{d\;V_{GS}}{d\log I_{DS}}$$

The *SS* was 131.9 and 94 mV/dec for the CTA and MWA processed a-IGZO TFTs, respectively. In addition, the interface trap density (*D_it_*) between the a-IGZO channel and the SiO_2_ gate insulator was extracted from *SS* using the following equation:

(3) $$D_{it}=\left(\frac{q\cdot SS\cdot \log\left(e\right)}{k_{B}T}-1\right)\frac{C_{ox}}{q}$$

where *q*, *k_B_*, and *T* are the elementary electric charge, Boltzmann’s constant, and absolute temperature, respectively. The *D_it_* was 3.7 × 10^12^ and 2.6 × 10^12^ cm^−2^ eV^−1^ for the CTA and MWA processed a-IGZO TFTs, respectively. This result reveals that MWA effectively improves the interface states of a-IGZO/SiO_2_, despite a processing time of just 2 min, which is much less than the 30 min for CTA.

**Figure 8:**
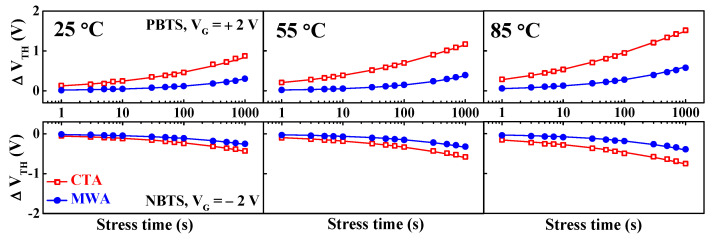
Time dependence of the V_TH_ shift under PBTS and NBTS tests at 25, 55, and 85 °C for CTA (300 °C, 30 min) and MWA processed (600 W, 2 min) a-IGZO TFTs on flexible PI substrates. The symbols represent the measured V_TH,_ and the lines represent the fitting curves using the stretched-exponential equation.

Figure 8 shows threshold voltage shift (∆V_TH_) when PBTS (V_G_ = V_TH0_ + 2 V, V_D_ = 0 V) and NBTS (V_G_ = V_TH0_ − 2 V, V_D_ = 0 V) are applied for 1000 s at a temperature of 25, 55 and 85 °C each, where V_TH0_ is the initial V_TH_ without gate stress. The driver TFTs in the pixel circuitry of a display are subjected to long-term positive or negative bias stress during pixel operation and stand-by. Consequently, the ∆V_TH_ against prolonged gate bias stress is one of the crucial factors in terms of the reliability of a device. In Figure 8, it is shown that the V_TH_ shifted in the positive or negative direction depending on the polarity of the electrical stress over all measured temperatures. Oxygen and moisture are more likely to be adsorbed onto the a-IGZO back-channel under positive gate bias stress, which results in a positive ∆V_TH_ by forming electron trapped acceptor-like trap states in the channel region [23,24]. By contrast, under negative gate bias stress, negative ∆V_TH_ is caused because of hole traps in oxygen vacancies that behave as donor-like trap states [25,26]. In addition, the reason why the threshold voltage shift in the NBTS is smaller than that in the PBTS is due to the fact that only a small number of holes in the a-IGZO channel have n-type conduction properties [27]. For both PBTS and NBTS, at all measurement temperatures, the ∆V_TH_ over stress time was smaller in magnitude and slower in MWA than in CTA. The ∆V_TH_ for thermally activated charge trapping can be fitted through the following stretched-exponential function for the stress time [28]:

(4) $$\Delta V_{\mathrm{TH}}=\Delta V_{O}\left\{1-\exp\left[-{\left(\frac{t}{\tau }\right)}^{\beta }\right]\right\}$$

where the V_O_ is the V_TH_ shift at infinite time, *β* is the stretched-exponential exponent, and *τ* is the characteristic charge trapping time. It is found that the fitting line agrees well with the experimental data and that τ depends on the annealing processes CTA and MWA.

**Figure 9:**
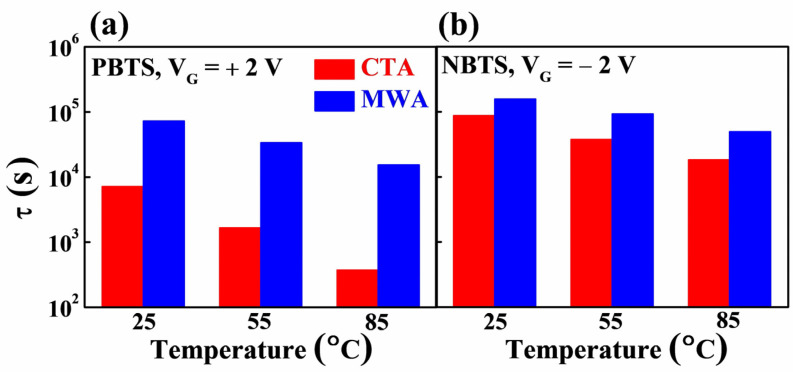
Comparison of charge trapping time *τ* of a-IGZO TFT on flexible PI substrate extracted by (**a**) PBTS and (**b**) NBTS test according to PDA method.

Figure 9 shows the charge trapping time *τ*, which is the time it takes for carriers to be trapped inside the insulator or at the channel/insulator interface of a-IGZO FETs, extracted from the time dependence of the ∆V_TH_ under PBTS and NBTS in Figure 8. It can be seen that the *τ* values extracted from the PBTS in Figure 9a are lower than those extracted from the NBTS in Figure 9b, indicating that the PBTS deterioration due to electron trapping is dominant in the a-IGZO TFTs. In terms of annealing methods, the *τ* value of MWA is higher than that of CTA. Accordingly, it can be concluded that MWA is a more effective PDA method than CTA to improve the electrical properties and stability of a-IGZO TFT.

**Figure 10:**
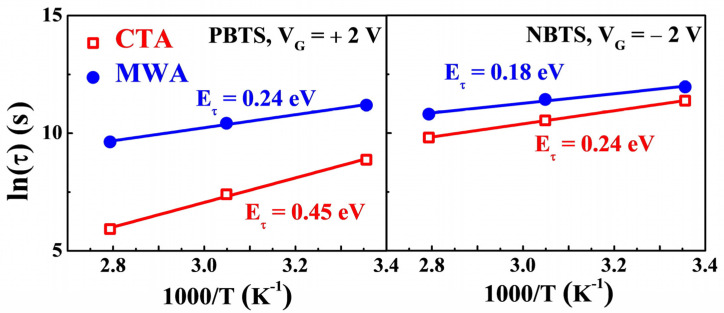
The ln(*τ*) as a function of reciprocal temperature (1/*T*) for CTA (300 °C, 30 min) and MWA processed (600 W, 2 min) a-IGZO TFTs on flexible PI substrates.

Figure 10 shows the plot of ln(*τ*) as a function of the reciprocal of temperature (1/*T*). In the stretched-exponential Equation (4), the charge trapping time *τ* of the thermally activated carriers is expressed as

(5) $$\tau =\tau_{0}\exp\left(\frac{E_{\tau }}{k_{B}T}\right)=\nu^{-1}\exp\left(\frac{E_{\tau }}{k_{B}T}\right)$$

where the *τ*_0_ and *ν* are the thermal pre-factor and frequency pre-factor for emission across the barrier, respectively. The thermal activation energy is given by E_a_ = *E_τ_β*, where *E_τ_* is the average effective energy barrier height for carrier transport. Because the *τ* decreases with an increase in temperature as shown in Figure 9, *E_τ_* can be extracted using Arrhenius relation and Equation (5). The extracted *E_τ_* was 0.24 and 0.18 eV for PBTS and NBTS for MWA TFTs, respectively. By contrast, in CTA TFTs, E_τ_ for PBTS and NBTS was 0.45 and 0.24 eV, respectively, higher than that of MWA TFTs. In some of the previous studies, it has been reported that lower *E_τ_* is due to the more ordered structure of the a-IGZO channel layer [29]. The smaller *E_τ_* in MWA TFTs than in CTA TFTs implies that the MWA processing results in a more ordered structure of the a-IGZO channel than the CTA processing. This is because MWA directly transfers microwave energy to the material and efficiently converts it into heat, enabling the formation of a more orderly structured channel layer despite a shorter process time than CTA.

**Figure 11:**
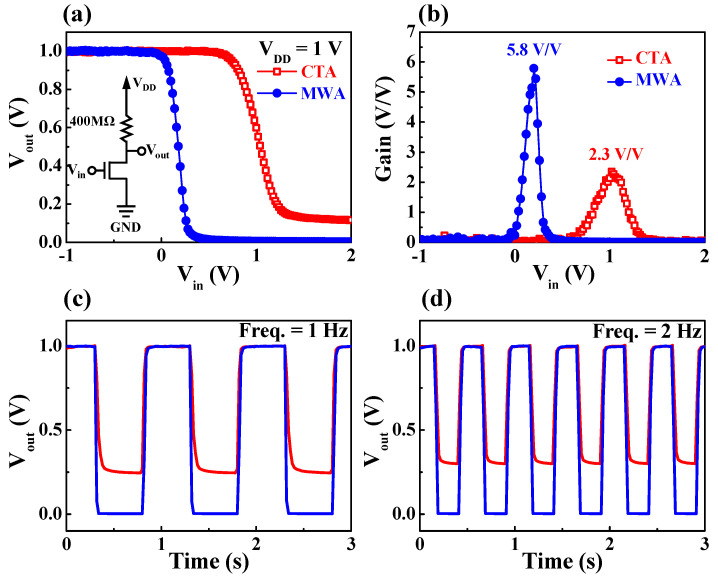
Resistive load inverter characteristics of CTA (300 °C, 30 min) and MWA processed (600 W, 2 min) a-IGZO TFTs on flexible PI substrates. (**a**) voltage transfer characteristics (VTC) curve. The inset is a schematic of an equivalent circuit for a resistive load inverter. (**b**) Voltage gain. Dynamic inverting responses at a frequency of (**c**) 1 Hz and (**d**) 2 Hz.

Figure 11 shows the static voltage transfer characteristics (VTC) and dynamic inversion characteristics of a resistive load inverter circuit constructed by connecting a load resistance of 400 MΩ in series with an a-IGZO TFT. For the application of TFTs as display driver circuits, it is necessary to evaluate the performance of the inverter, which is an elementary building block of digital devices. Figure 11a shows typical VTC curves of a resistive load inverter versus the DC gate input voltage (V_in_). As shown in the equivalent circuit inset in Figure 11a, a constant voltage of 1 V was applied to the resistor connected in series with the drain while the source of the a-IGZO TFT was grounded. It can be seen that the voltage states of the output and input of inverters composed of both CTA TFT and MWA TFT are clearly inverted. In the case of the MWA TFT-based inverter, the output state “1” is maintained until the input voltage (V_in_) reaches −0.04 V, where it transitions to the “0” at V_in_ = 0.46 V. However, the CTA TFT-based inverter keeps the output state at “1” until V_in_ reaches 0.56 V, and the output transitions to the “0” at V_in_ = 1.36 V, resulting in slower switching properties. In addition, the output voltage (V_out_) corresponding to the “0” state was 0.0 and 0.121 V for MWA and CTA, respectively. Figure 11b shows the voltage gain −dV_out_/dV_in_, where the maximum gain of the MWA TFT-based inverter (5.8 V/V) is more than two times higher than that of the CTA TFT-based inverter (2.3 V/V). Figure 11c,d show the dynamic inverting responses for AC input voltages with frequencies of 1 and 2 Hz, respectively. The MWA TFT-based inverter displayed ideal dynamic characteristics in which the output state is the inverted form of the input signal. Moreover, it is found that the CTA TFT-based inverter with low mobility has a relatively higher voltage in the “0” state than the MWA TFT-based inverter due to the response delay. This difference in dynamic inverting response (0.25 V at 1 Hz and 0.30 V at 2 Hz) is more pronounced at higher input frequencies. The “0” state voltage and higher frequency response speed of an MWA TFT-based inverter reduces power consumption and increases operating speed in practical display driver circuit applications.

## 4. Conclusions

We proposed the fabrication of a-IGZO TFTs without thermal damage on a flexible and transparent PI substrate by applying MWA with efficient energy conversion. Prior to device fabrication, MWA or CTA was applied to a transparent and flexible PI substrate, and thermal damage was evaluated through optical transmittance and bending endurance tests. The PI substrate did not suffer any damage even at the high microwave power in MWA, whereas it suffered significant damage at the high temperatures in CTA. Therefore, MWA is an efficient thermal damage-free process with material-selective heating, enabling the fabrication of high-performance a-IGZO TFTs on flexible PI substrates. Meanwhile, the flexible a-IGZO TFTs were fabricated under two PDA conditions: MWA (600 W for 2 min) and CTA (300 °C for 30 min) with the same sample heating temperatures, and its electrical properties and reliability were evaluated. It was demonstrated that MWA effectively improves the electrical properties of TFTs such as μ_FE_, *SS*, *D_it_* and *I_on_/I_off_* in spite of consuming less processing time as compared to CTA. In addition, MWA reduces the threshold voltage shift for PBTS and NBTS tests under the same measurement temperature, resulting in improved stability of a-IGZO TFTs. The extracted charge trapping time (*τ*) and the effective energy barrier height (*E_τ_*) for charge transport from the time dependence of ∆V_TH_ in PBTS and NBTS tests under various measurement temperatures revealed that MWA improves device reliability by configuring a more orderly channel structure than CTA. Comparing the switching performance of configuring resistive load inverter circuits, the MWA TFT-based inverter showed better VTC curve, gain, and dynamic switching characteristics than the CTA TFT, resulting in less power consumption and faster switching speed in actual device applications. In conclusion, thermal damage-free MWA, which is characterized by high efficiency, low cost, and high process compatibility is expected to be a promising technology for flexible display applications.

## Data Availability

The data presented in this study are available from the corresponding author upon reasonable request.
